# The Effects of Micro-vessel Curvature Induced Elongational Flows on Platelet Adhesion

**DOI:** 10.1007/s10439-021-02870-4

**Published:** 2021-10-19

**Authors:** Christian J. Spieker, Gábor Závodszky, Clarisse Mouriaux, Max van der Kolk, Christian Gachet, Pierre H. Mangin, Alfons G. Hoekstra

**Affiliations:** 1 Computational Science Lab, Faculty of Science, Institute for Informatics, University of Amsterdam, Amsterdam, The Netherlands; 2 Université de Strasbourg, INSERM, EFS Grand-Est, BPPS UMR-S 1255, FMTS, Strasbourg, France

**Keywords:** Non-trivial vessel geometry, Blood rheology, Cell free layer, Cell-resolved simulation, Elongational flow

## Abstract

The emerging profile of blood flow and the cross-sectional distribution of blood cells have far reaching biological consequences in various diseases and vital internal processes, such as platelet adhesion. The effects of several essential blood flow parameters, such as red blood cell free layer width, wall shear rate, and hematocrit on platelet adhesion were previously explored to great lengths in straight geometries. In the current work, the effects of channel curvature on cellular blood flow are investigated by simulating the accurate cellular movement and interaction of red blood cells and platelets in a half-arc channel for multiple wall shear rate and hematocrit values. The results show significant differences in the emerging shear rate values and distributions between the inner and outer arc of the channel curve, while the cell distributions remain predominantly uninfluenced. The simulation predictions are also compared to experimental platelet adhesion in a similar curved geometry. The inner side of the arc shows elevated platelet adhesion intensity at high wall shear rate, which correlates with increased shear rate and shear rate gradient sites in the simulation. Furthermore, since the platelet availability for binding seems uninfluenced by the curvature, these effects might influence the binding mechanics rather than the probability. The presence of elongational flows is detected in the simulations and the link to increased platelet adhesion is discussed in the experimental results.

## Introduction

The effects of curved vessel geometry on blood flow were investigated thoroughly from a macroscopic viewpoint,^2,31^ where blood is approximated as a continuum fluid and the biological implications (often in connection to cardiovascular diseases) are commonly linked to the magnitude or inhomogeneity of wall shear stress. On the level of smaller, micron-scale vessels, or when investigating near-wall processes, the continuum description is no longer sufficient. The continuum approximation can lead to several-fold differences in shear rate and shear stress close to the wall.^30^ The complex nature of blood as a fluid is dictated primarily by its physiological composition of blood plasma and immersed deformable cells. These cellular components account for approximately half of the volume fraction. The hematocrit value, corresponding to the red blood cell (RBC) concentration, is around 44% in healthy humans. Moreover, blood contains less numerous cells (e.g. platelets (PLTs) and white blood cells (WBCs)) that account for about 1% in total blood volume.^3^

Due to these cellular components blood behaves as a non-Newtonian fluid with unique rheological properties in the confined geometry of blood vessels, giving rise to a multitude of phenomena, such as the Fåhræus and Fåhræus-Lindqvist effects. These two effects occur as a consequence of the formation of the $\hbox {red blood cell free layer (CFL)}$, which in turn is caused by the lift force and shear flow induced axial migration of $\hbox {RBCs}$.^22^ The $\hbox {CFL}$ acts as a lubrication layer for the bulk of cellular flow due to the locally reduced blood viscosity.^17^ As frequently discussed in literature, an increased hematocrit value results in a smaller $\hbox {CFL}$ width and an increase in flow velocity has the opposite effect, due to a larger lift force.^9,29^ $\hbox {PLTs}$ undergo radial migration towards the vessel wall and into the CFL.^18^ This process, called margination, creates an increased availability of $\hbox {PLTs}$ close to the vessel wall. Zydney and Colton attributed this marginating behaviour to diffusion gradients,^35^ while recently Závodszky *et al*. showcased a strong dependency on the hematocrit gradient.^34^ Kotsalos *et al*. proposed that a Lévy flight solution fits the mean-field description of the resulting motion of $\hbox {PLTs}$.^10^ The cellular flow dynamics have far reaching biological implications in various diseases, for instance in the oxygenation of tumor tissues,^4^ or in the margination process in the presence of stiffened diabetic cells.^6^ One particular process of importance that is influenced is the adhesion of $\hbox {PLTs}$. This process, which occurs both in physiological hemostasis, as well as pathological thrombosis, is found to be highly shear dependent and sensitive to hydrodynamic alterations.^11,19^ The shift of initially even shear gradients, e.g. caused by a sudden reduction in vessel diameter (vasoconstriction), leads to the presence of so called elongational flows. These flow fields, defined by exerting tensile forces, are found to promote $\hbox {PLT}$ adhesion under certain conditions, enabled by the mediation of prominent plasma molecule $\hbox {von Willebrand factor (vWF)}$.^13,23^ Furthermore, $\hbox {PLT}$ adhesion is known to depend on the presence of a $\hbox {CFL}$^29^ as well as the level of hematocrit.^24^ The effects of these essential blood flow parameters on $\hbox {PLT}$ adhesion were investigated under static flow conditions, in straight geometries.

Here, the effects of curvature are investigated by simulating the cellular movement of $\hbox {RBCs}$ and $\hbox {PLTs}$ in a half-arc channel for multiple wall shear rate and hematocrit values. The simulations show significant differences in the emerging shear rate values and distributions between the inner and outer arc of the channel curve, while the cell positions remain predominantly unaffected. The simulation predictions are also compared to experimental $\hbox {PLT}$ adhesion in a similar curved geometry. The changes in the shear-rate patterns, inducing the presence of elongational flow, correlate to the location of changes in the $\hbox {PLT}$ adhesion intensity. The main focus is on the accurate simulation and evaluation of the flow conditions, $\hbox {CFL}$, and cell distributions. However, a comparison to *in vitro* assays in a similar curved microfluidic geometry is presented as well and the possible implications of elongational flow in vessel curvature on $\hbox {PLT}$ adhesion are discussed.

## Materials and Methods

### Experimental Setup: *In Vitro* Flow-Based Studies

**Figure 1:**
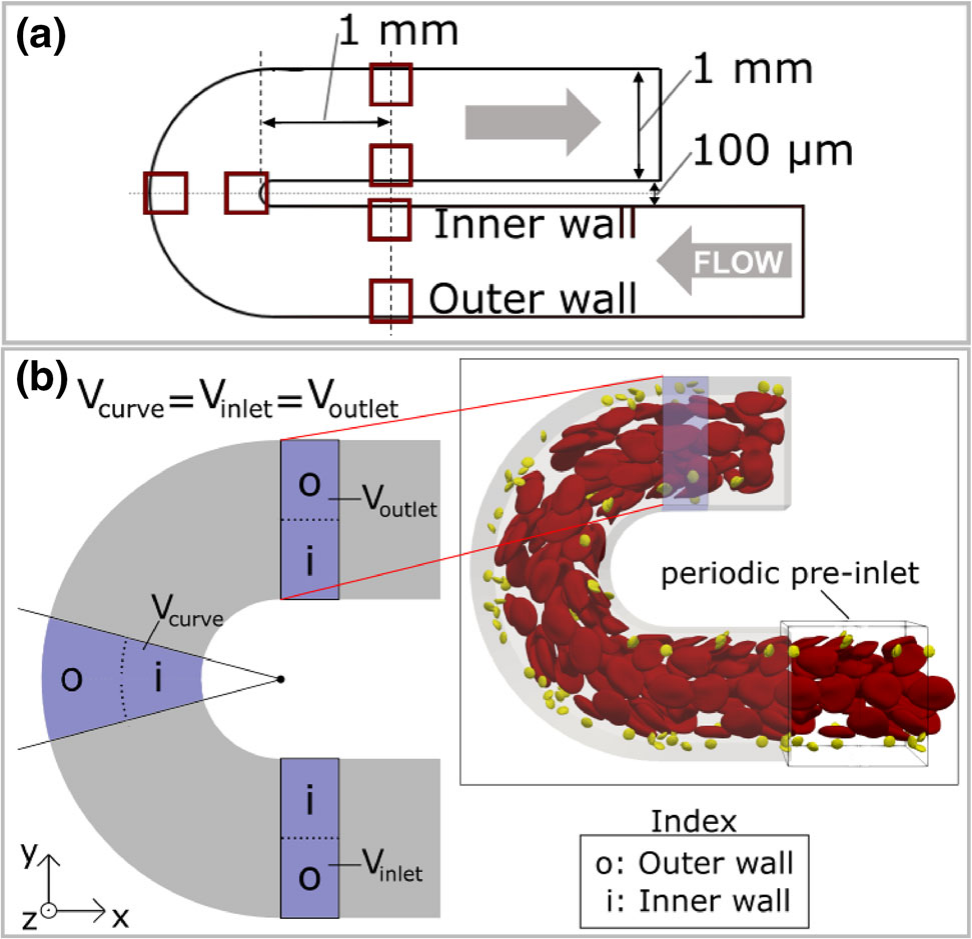
Experimental and simulation setup. (a) Setup of curved $\hbox {MFD}$ blood experiments with highlighted regions of interest in red. (b) Setup of curved channel domain with cross-sectional dimensions of $25 \times 25$*μ*m^2^. Driving force is set in negative *x*-direction inside the periodic pre-inlet (see top right). The regions of interest with equal volume (*V*_inlet_, *V*_curve_ and *V*_outlet_) are highlighted in the top view of the channel (see left), where the corresponding inner and outer wall division used in the cell distribution evaluation is marked with the letters o and i, respectively.

Experiments using flow-based assays are performed in the same manner as previously described by Receveur *et al*.^14^ The polydimethylsiloxane (PDMS)-based microfluidic device (MFD) has a square duct channel with a cross section of $1 \times 1$ mm^2^ and the diameter of the inner curve is 100 *μ*m (see Fig. 1a), creating a $180^\circ$ angle U-turn. The channel is coated overnight at $4\,^\circ {\hbox {C}}$ with type I fibrillary collagen (200 *μ*g/mL) before being blocked with $\hbox {human serum albumin (HSA)}$ in 1% $\hbox {phosphate buffered saline (PBS)}$ for 30 min at room temperature. Hirudinated ($> 100$ U/mL) human whole blood is perfused at $37\,^\circ \hbox {C}$ through the U-shape channels at volumetric flow rates of 3 and 16 mL/min using a programmable syringe pump (PHD 2000, Harvard Apparatus, Holliston, MA, USA). Assuming a continuous Newtonian fluid these flow rates result in $\hbox {wall shear rates (WSRs)}$ of 300 and 1600 $\hbox {s}^{-1}$, respectively, at the midpoint of the wall edges in the straight inlet. Previous work, e.g. by van Rooij *et al*.,^29^ has shown that this assumption is not accurate for cellular blood flow and leads to wrong $\hbox {WSRs}$. However, as it is still common practice in experimental work, it is adopted here as well. A vascular $\hbox {WSR}$ of 300 $\hbox {s}^{-1}$ occurs in the scale of conduit arteries, such as the carotid, as well as venules. Larger $\hbox {WSRs}$ in the range of 1600 $\hbox {s}^{-1}$ can be observed in the smallest arteries of the vasculature, the arterioles.^16^ The values are chosen to cover a wide range of physiological shear rates.

In some sets of experiments, hirudinated whole blood is preincubated with a Fab fragment of a blocking anti-GPIb$\upalpha$ antibody (10 *μ*g/mL), named ALMA12, for 15 min at 37 $^\circ \hbox {C}$ before perfusion.

$\hbox {PLT}$ adhesion is visualized at the regions of interest (see Fig. 1a) using $\hbox {differential interference contrast (DIC)}$ microscopy images obtained with an inverted Leica DMI 4000 B microscope (Leica Microsystems, Mannheim, Germany) coupled to a complementary metal-oxide semiconductor (CMOS) camera (ORCA-Flash4.0 LT, Hamamatsu, Massy, France). The area of the thrombi is determined by utilising the Image J software (National Institutes of Health) to automatically delineate the surface of the thrombi which is expressed in *μ*m^2^. The results are then quantified as thrombi coverage fraction of the observed area.

### Simulation Setup

The *in silico* experiments are based on the open-source cell-resolved blood flow model HemoCell,^33^ which consists of a $\hbox {lattice Boltzmann method}$ based fluid solver for the incompressible blood plasma and a $\hbox {discrete element method}$ membrane solver for the cell deformation mechanics. These two components are coupled by the $\hbox {immersed boundary method}$. Both the membrane mechanical models of $\hbox {RBCs}$ and $\hbox {PLTs}$ as well as the bulk flow rheology of the entire model have been thoroughly validated for both single cell and bulk flow dynamics.^32–34^

To simulate cellular blood flow in a U-shaped channel geometry the same model parameters are used as in the validation studies.^32–34^ The cross-section of the channel is a square duct, following the current methods in experimental platelet adhesion assays^12,30^ to allow comparison with the microfluidic experimental results. The width of the channel is $25 \times 25$ *μ*m^2^ and the inner diameter of the curved annulus section is also set to 25 *μ*m (see Fig. 1b). To allow for comparison with the experimental results, the simulated flow rate is setup to result in $\hbox {WSRs}$ of ${\dot{\gamma }}$ = 300 $\hbox {s}^{-1}$ and ${\dot{\gamma }}$ = 1600 $\hbox {s}^{-1}$, respectively, at the midpoint of the wall edges in the straight inlet of the simulated channel using the same continuous flow assumption as in the experimental setup. The cells are randomly distributed in the inflow domain to result in a discharge hematocrit of 30%. The $\hbox {PLT}$ concentration is fixed to $900,000\, {\mathrm{PLT}}/{\mu \mathrm{L}}$, which is larger than physiological levels (150,000–$400,000\, {\mathrm{PLT}}/{\mu \mathrm{L}}$).^3^ This allows for increased statistical significance in evaluating the results, while still being dilute enough to avoid influencing the overall cellular flow dynamics.

The continuous inflow boundary condition for cells (denoted as periodic pre-inlet in Fig. 1b) is implemented according to Azizi *et al*.^26^ Note that this periodic pre-inlet boundary domain mimicks an infinitely long straight channel, ensuring that the incoming cell distributions are fully developed at the point of entry to the main U-shape domain. All simulations are executed on the Cartesius supercomputer (SURF, Amsterdam, Netherlands; https://userinfo.surfsara.nl/systems/cartesius).

### Evaluation Method

To determine the $\hbox {CFL}$ width in the simulated results, it is defined as the distance from the wall where the $\hbox {CFL}$ volume fraction reaches a threshold value of 5%. To simplify the calculation it is solely based on the center of mass of each $\hbox {RBC}$, neglecting the rotated and deformed membrane volume.

The curved channel domain is evaluated at three different positions along the flow direction both on the inner and on the outer side of the channel. These locations are situated in the middle of the curved region, right before, and right after the curvature in the straight ’inlet’ and ’outlet’ sections, as shown in Fig. 1b. The ’curved’ section is defined as an annulus sector with a $60^\circ$ angle. The volume of the evaluated ’inlet’ and ’outlet’ sections equals the volume of the ’curved’ section. To calculate the $\hbox {CFL}$ and the overall cell distribution in each section, the cell position coordinates along the flow are projected onto the center line of the channel. For the cell distribution evaluation, each section volume is divided into an inner and outer wall layer counterpart (see Fig. 1b). This results in a visualisation of an accumulated cell concentration per layer volume. The inner wall refers to the half of each section situated at the inner arc of the curve and the outer wall respectively at the outer arc. This separation allows to evaluate average behavior in the layers which reduces statistical noise.

To localise and quantify elongational flows within the domain, the rate of elongation ${\dot{\varepsilon }}$ is calculated for the planar profile. From the rate of strain tensor, given by:

1 $$\begin{aligned} e_{ij} = \begin{bmatrix} e_{11} &{} e_{12} &{} e_{13} \\ e_{21} &{} e_{22} &{} e_{23} \\ e_{31} &{} e_{32} &{} e_{33} \end{bmatrix} \end{aligned}$$

the magnitude of the diagonal elements in flow dimensions ($e_{11}$ and $e_{22}$ for *x* and *y*) across the center *z*-plane of the domain is calculated. This results in the magnitude of elongation, i.e. the rate of elongation:

2 $$\begin{aligned} {\dot{\varepsilon }}=\sqrt{e_{11}^2+e_{22}^2} \end{aligned}$$

displayed in s^−1^.

## Results

### *In Vitro* Results

**Figure 2:**
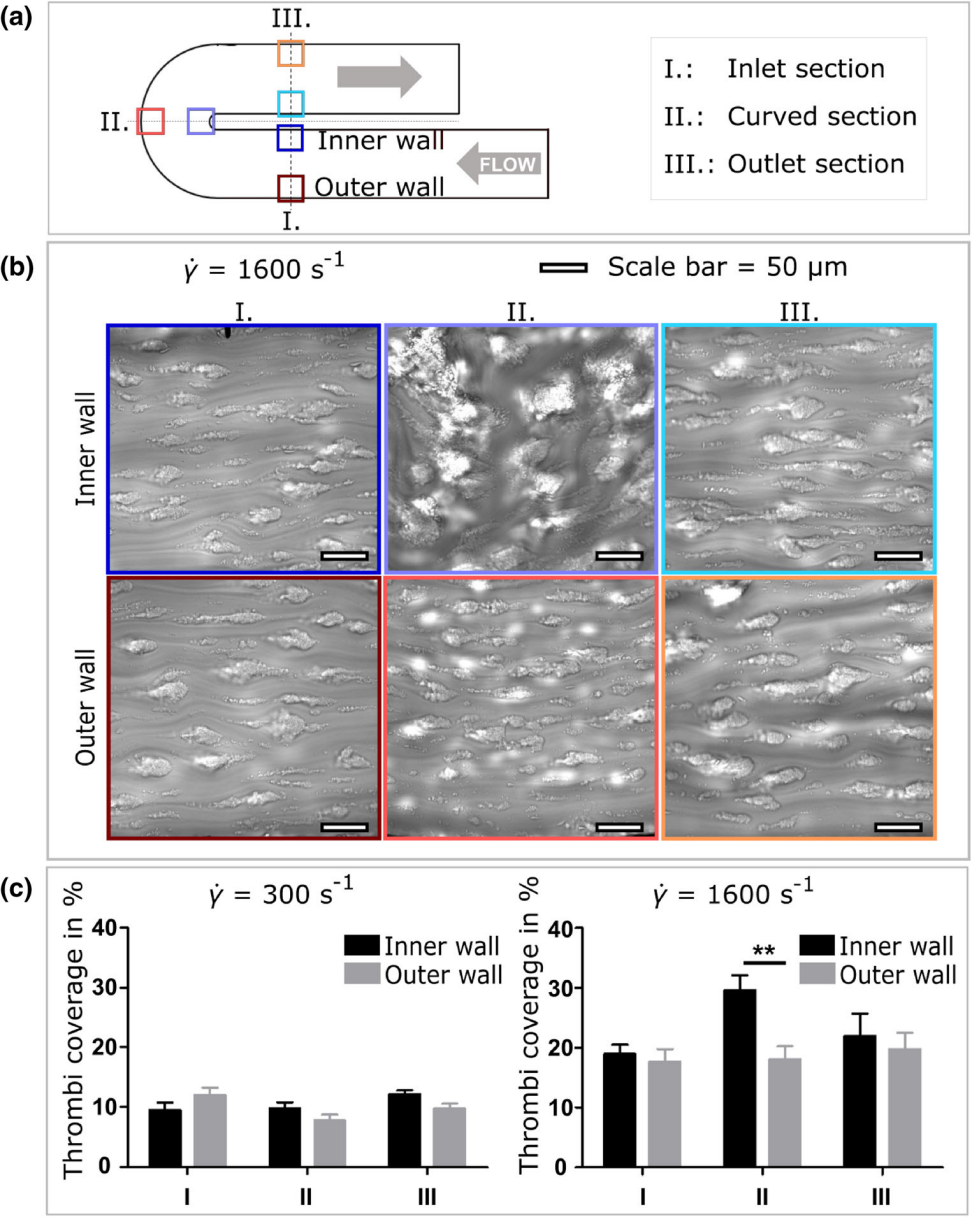
Impact of flow generated in a curved section on human PLT aggregation to collagen. Hirudinated human whole blood is perfused through channels of a $\hbox {MFD}$ coated with a solution of type I fibrillar collagen (200 *μ*g/mL). (a) Schematic and dimensions of the microfluidic “U-shaped” channel. The color-coded squares indicate the 0.3 mm^2^ regions of interest observed by video-microscopy. (b) Representative $\hbox {DIC}$ images of $\hbox {PLT}$ aggregate formation at inner and outer arc on immobilized collagen at 1600 s^−1^ after 4 min. Scale bar: 50 *μ*m. The frame color refers to the color-coded positions in (a) and the numbered columns to the labeled inlet, curved and outlet section in (a). (c) The quantified surface coverage of platelet aggregates obtained after 4 min of perfusion at ${\dot{\gamma }}$ = 300 s^−1^ and ${\dot{\gamma }}$ = 1600 s^−1^. The bars indicate the mean ± SEM thrombi coverage in the 6 highlighted regions of 5 separate experiments performed with different blood donors.

To study the impact of altered hemodynamic conditions generated in a curved vessel geometry on $\hbox {PLT}$ function, the thrombi coverage on the described collagen coated $\hbox {MFD}$ surface is evaluated after blood perfusion. Real-time video-microscopy based on $\hbox {DIC}$ imaging indicates that $\hbox {PLTs}$ adhere efficiently in all regions observed and form large aggregates (Fig. 2b). The color-coded regions on the sketch in Fig. 2a mark the position of the framed microscopic images in Fig 2b. At the $\hbox {WSR}$ of 1600 s^−1^ in the region of the inner wall of the curved section (II.) the $\hbox {PLT}$ aggregates display a different orientation as compared to the five other regions of interest which show a clear orientation in flow direction (Fig. 2b). Furthermore, the aggregates appear much larger in this region (inner wall II.) compared to the other ones. This is confirmed by the fractional surface coverage of the thrombi being significantly increased in the inner section of the curved region (II.) by 10% to a total of 30% compared to the outer region and to the evenly covered inlet section (I.) (Fig. 2c). The effect subsides into the outlet section (III.) with no significant difference outside of the increased $\hbox {standard error of the mean (SEM)}$ error margin compared to the inlet section. In contrast, the lower shear (${\dot{\gamma }}$ = 300 s^−1^) experiment exhibits no significant difference in thrombi coverage between inner and outer surface layer at any of the three sections as shown in Fig. 2c.

### *In Silico* Results

**Figure 3:**
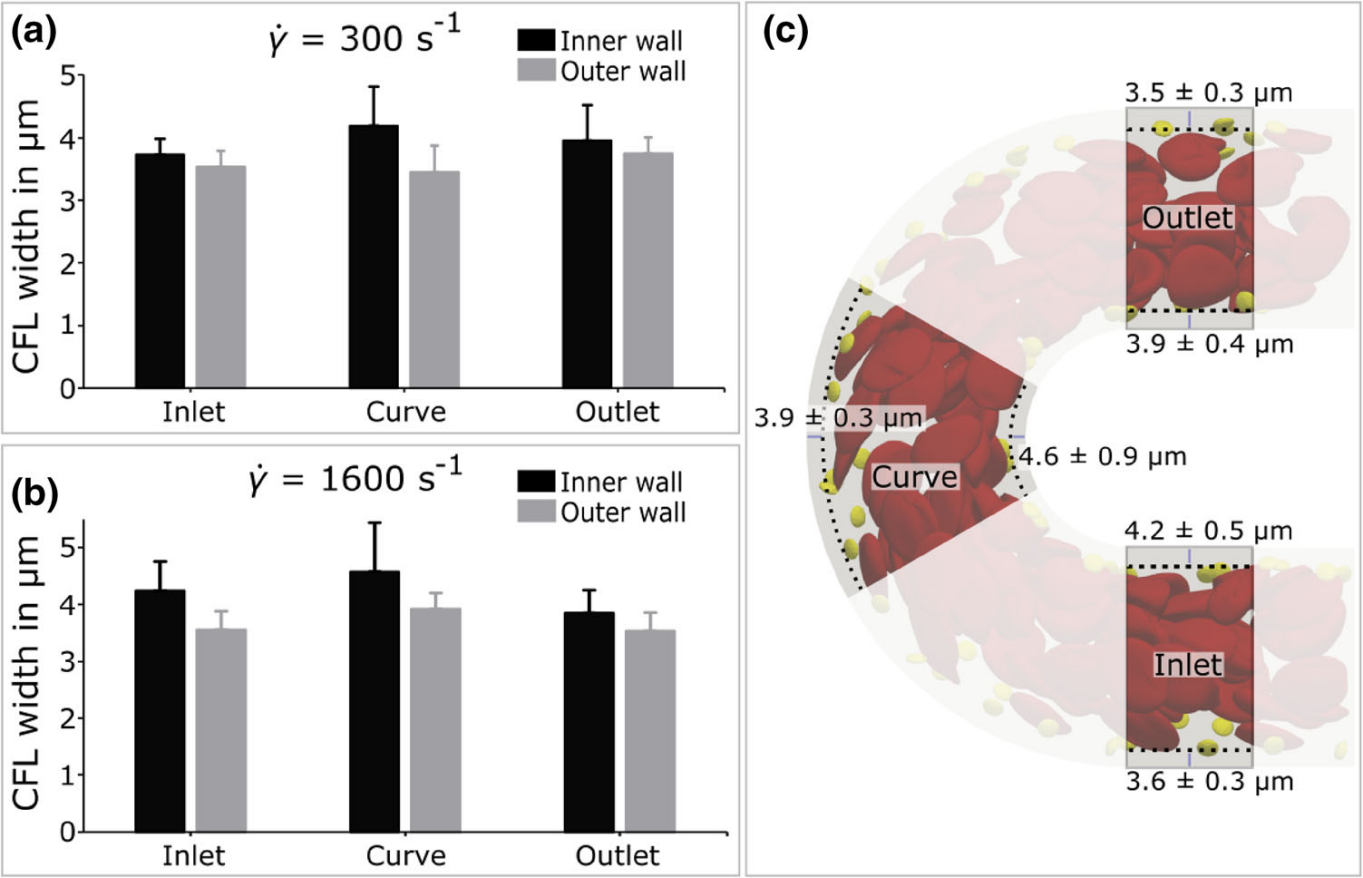
CFL width in silico results. (a) & (b) CFL width results at inner and outer wall of the inlet, curve and outlet section for the H = 30% simulations at $\hbox {WSRs}$ ${\dot{\gamma }}$ = 300 s^−1^ and ${\dot{\gamma }}$ = 1600 s^−1^, respectively. (c) Visual representation of uniform $\hbox {CFL}$ width in 30$\hbox {WSR}$ of 1600 s^−1^ simulation at inner and outer wall in the respective sections after 1 s of flow with marginated $\hbox {PLTs}$. The results are averaged between 0.9 and 1 s in 0.001 s time windows.

To determine the root cause of the exhibited difference in $\hbox {PLT}$ adhesion in a curved vessel at physiological shear flow, the *in silico* experiments are evaluated with special emphasis placed on assessing deviations between inner and outer wall of the curvature. For the $\hbox {CFL}$ width this is achieved by comparing the average value ± $\hbox {standard deviation (SD)}$ at the inner and outer wall in the inlet, curved and outlet section. The results, summarised in Figs. 3a and 3b, display evidently the influence of (initial) $\hbox {WSR}$ on the $\hbox {CFL}$ width. As visualised in Fig. 3c, the results do not expose a significant influence of the curvature on $\hbox {CFL}$ width when taking the $\hbox {SD}$ range into account. This proves true for each performed simulation (see Figs. 3a and 3b).

Tateishi *et al*. performed blood experiments with similar 30% hematocrit in a straight blood vessel at a comparable diameter of 23.5 *μ*m. The measured $\hbox {CFL}$ width of 2.2 *μ*m is in the same order of magnitude as the simulated average width of 3.9 ± 0.4 *μ*m. The difference could be attributed to the simplified center of mass based $\hbox {RBC}$ localisation in the simulation evaluation as well as geometrical disparities (e.g. square duct channel vs. straight tubular vessel).^27^ The $\hbox {WSR}$ increase from ${\dot{\gamma }}$ = 300 s^−1^ to ${\dot{\gamma }}$ = 1600 s^−1^ does not lead to a significant difference in $\hbox {CFL}$ width.

**Figure 4:**
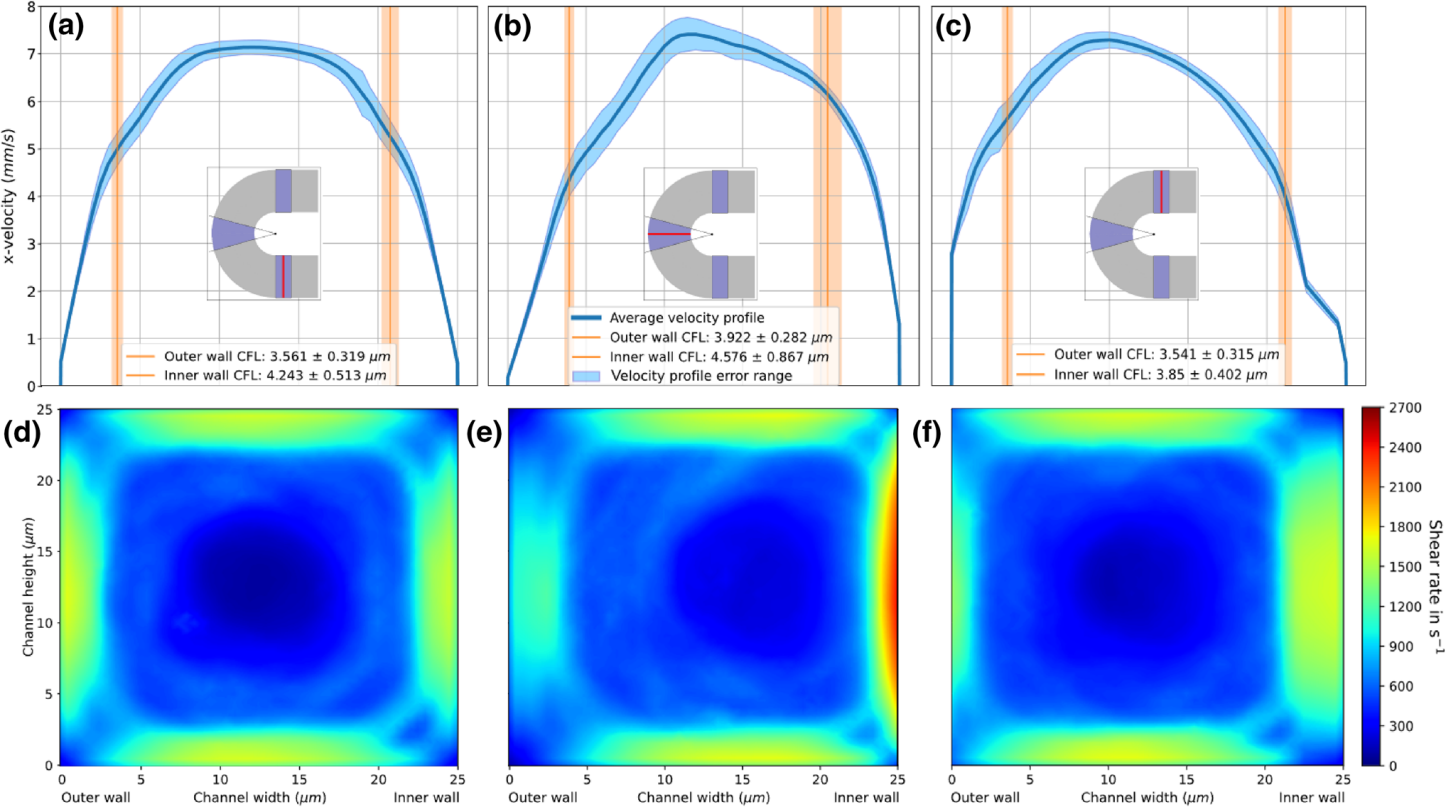
Flow profiles at ${\dot{\gamma }}$ = 1600 s^−1^. (a)–(c) 1D velocity profiles of 30% hematocrit and $\hbox {WSR}$ of 1600  s^−1^ simulation at the center line of the inlet, curved and outlet section, respectively (indicated by the red line in the layout sketch) at half the channel height. (d)–(f) Cross-sectional shear rate profiles at inlet, curved and outlet section, respectively. The positions are indicated by the red line in the layout sketches from (a)–(c). Results are averaged between 0.2 and 1 s.

While the $\hbox {CFL}$ width does not differ between the inner and outer wall in a statistically significant way in any of the sections, a substantial effect of the geometry curvature on the cross-sectional flow distribution is observed, here characterised by the respective 1D velocity and 2D shear rate profiles. Figure 4 highlights this effect by revealing a shift of the initially evenly blunted flow profiles towards the inner wall at the curved section, which is carried on into the outlet section. As an implication, the $\hbox {WSR}$ which is set to 1600 s^−1^ in the straight inlet, shifts in the curved section to a peak of larger than 2600 s^−1^ at the inner wall and around 1200 s^−1^ at the outer wall. While not as pronounced, this effect is carried on into the outlet section as well.

**Figure 5:**
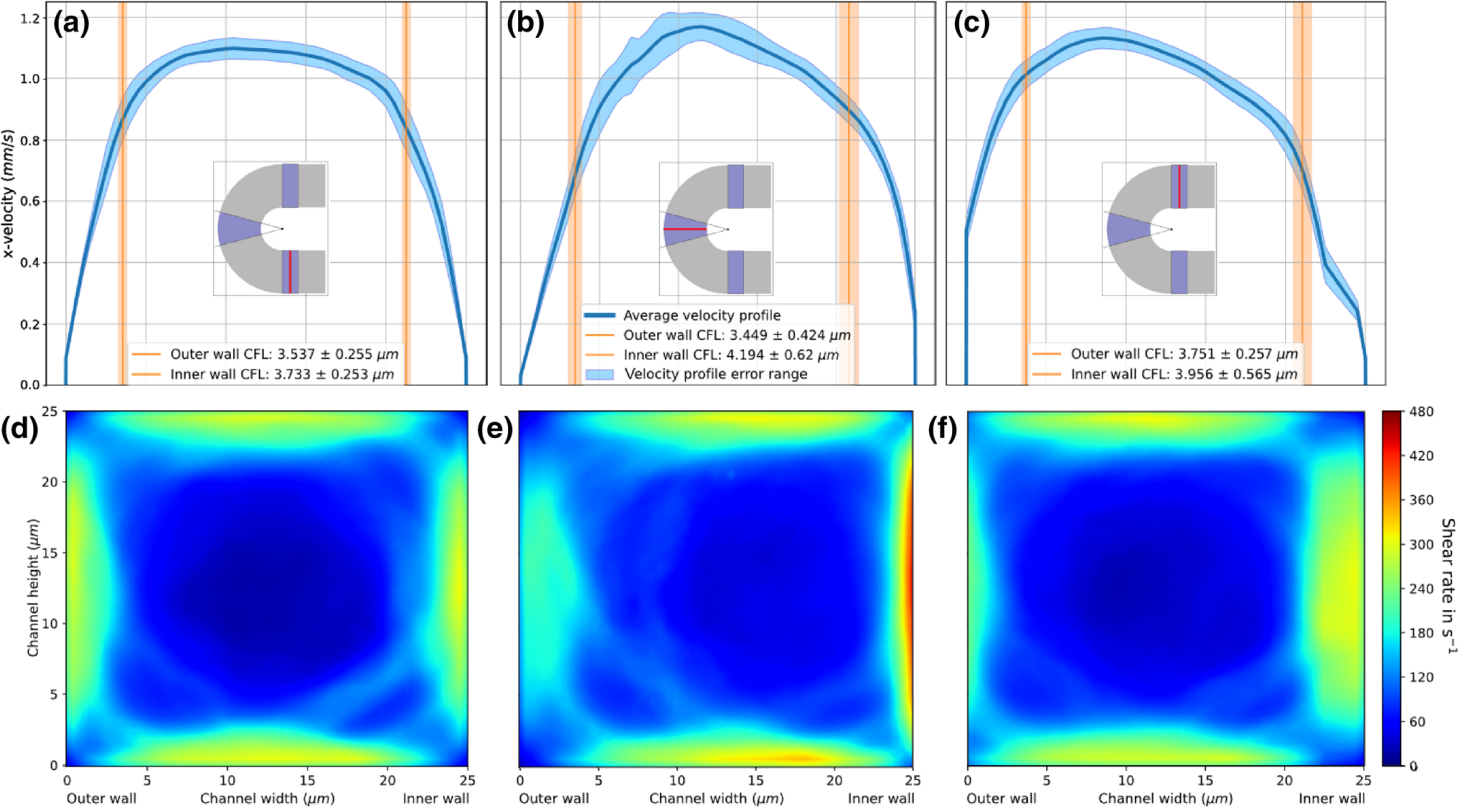
Flow profiles at ${\dot{\gamma }}$ = 300 s^−1^. (a)–(c) 1D velocity profiles of 30% hematocrit and $\hbox {WSR}$ of 300 s^−1^ simulation at the center line of the inlet, curved and outlet section, respectively (indicated by the red line in the layout sketch) at half the channel height. (d)–(f) Cross-sectional shear rate profiles at inlet, curved and outlet section, respectively. The positions are indicated by the red line in the layout sketches from (a)–(c). Results are averaged between 0.2 and 1 s.

The lower shear rate simulation with the same discharge hematocrit of 30% and an initial $\hbox {WSR}$ of 300 s^−1^ (see Fig. 5) deviates with roughly the same 2:1 ratio between the inner and outer wall at the center of the curved section, with a shear rate of over 480 s^−1^ at the inner wall and around 240 s^−1^ at the outer wall.

The inlet velocity profiles of both shear flow cases (Figs. 4a and 5a) display a dampened parabolic flow profile resembling the typical plug-shape caused by the high cell density at the center of the channel. The horizontal lines on Figs. 4a–4c and 5a–5c indicate the respective averaged $\hbox {CFL}$ width ± $\hbox {SD}$, clearly aligning with a step in the velocity profile. This step is caused by the transition from low local viscosity at the wall to higher viscosity in the $\hbox {RBC}$ rich bulk flow.

**Figure 6:**
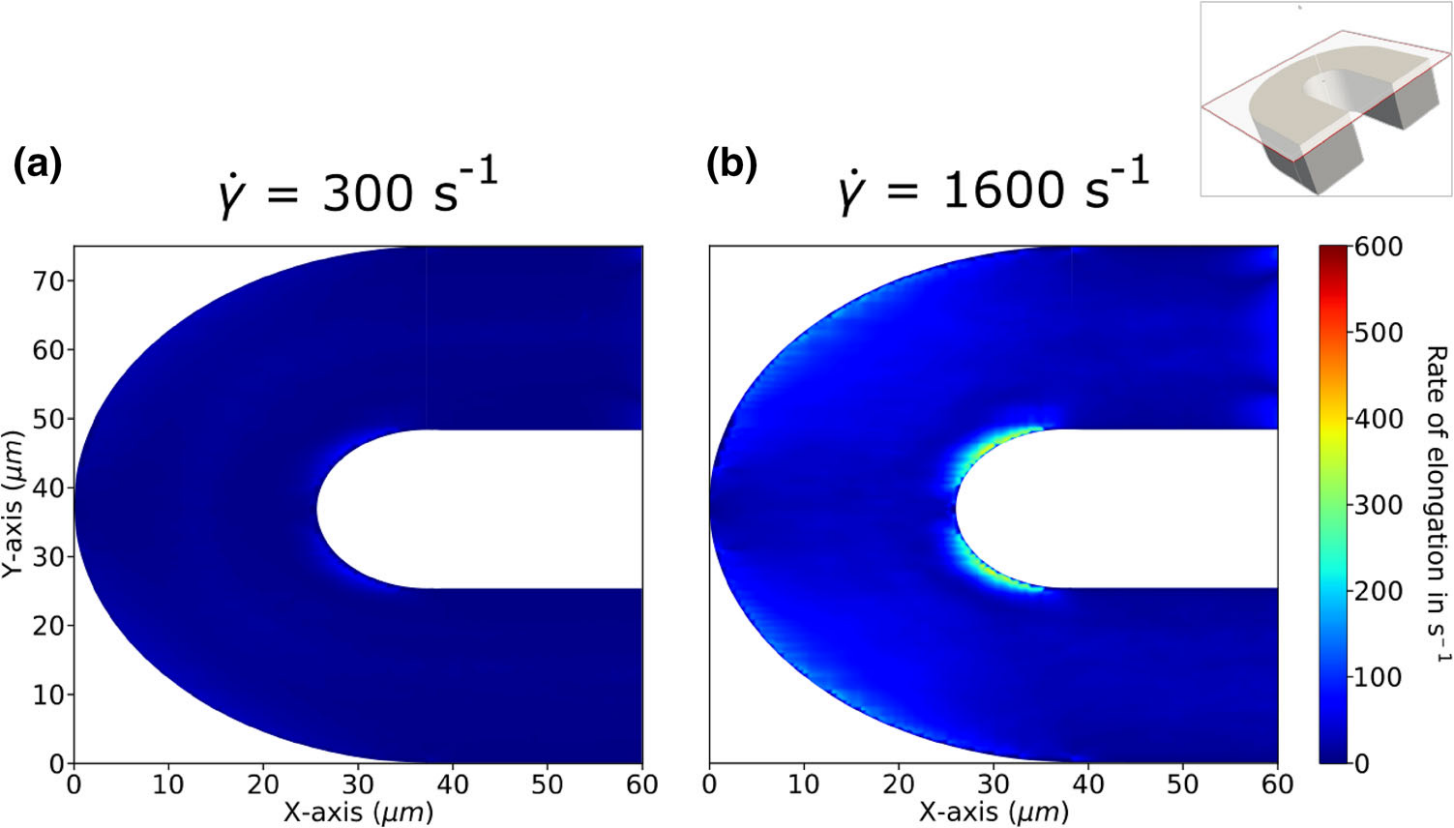
Top view of elongational flow magnitude accross channel. *Z*-plane of the domain with magnitude of the diagonal elements of the rate of strain tensor in flow (*x*- and *y*-) dimensions, resulting in a 2D elongation profile of the (a) 300 s^−1^ and (b) 1600 s^−1^ $\hbox {WSR}$ simulation. The plane is situated 1 *μ*m below the top boundary of the geometry in *z*-direction, as depicted in the top right inset panel. All results are averaged between 0.2 and 1 s.

Furthermore, by compressing the shear rate profile towards the inner wall, a shift in shear rate gradient distribution is induced as well, which is especially visible at the top and bottom of the channel of plot E in both Figs. 4 and 5. This shift in shear gradient naturally causes the occurrence of elongational flow fields. Figure 6 presents the rate of elongation across the plane of the domain. The plane is situated 1 *μ*m below the top boundary of the geometry in *z*-direction, which is within the same layer observed by the microscope in the experimental flow chamber. While both $\hbox {WSR}$ simulations show the highest rate of elongation ${\dot{\varepsilon }}$ right at the inner arc of the curve, the ${\dot{\gamma }}$ = 1600 s^−1^ case reaches significantly higher values of around ${\dot{\varepsilon }}$ = 379 s^−1^, compared to a peak value of ${\dot{\varepsilon }}$ = 71 s^−1^ in the ${\dot{\gamma }}$ = 300 s^−1^ case.

**Figure 7:**
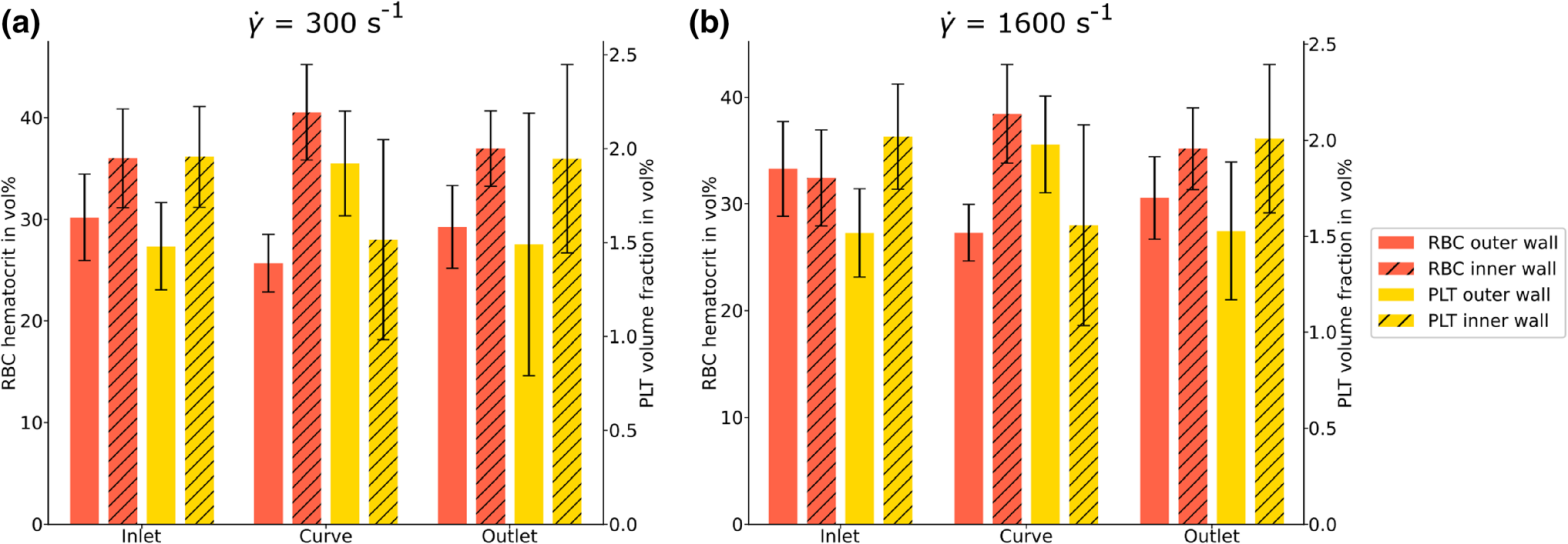
Cell distributions. Hematocrit (in red) and $\hbox {PLT}$ volume fraction (in yellow) distribution between inner (striped) and outer wall (blank) at initial $\hbox {WSRs}$ of (a) 300 s^−1^ and (b) 1600 s^−1^ for inlet, curve and outlet, respectively. All cell distribution results are averaged between 0.2 and 1 s.

In order to evaluate to what extent these shifted flow profiles influence the transportation of $\hbox {RBCs}$ and $\hbox {PLTs}$, the cell distribution in the regions of interest is investigated as an accumulated cell concentration per inner or outer wall layer volume averaged over time. As Figs. 7a and 7b shows, the $\hbox {RBC}$ distribution in the curved section exhibits a shift of around 10% towards the inner wall for both the high and low flow velocity case. Since the $\hbox {CFL}$ width did not change between the inner and outer wall in any section, it can be concluded that this observed shift is taking place in the center of the channel where the bulk of $\hbox {RBCs}$ is concentrated.^1^ Taking the $\hbox {SDs}$ into account, all remaining inner to outer wall comparisons in Fig. 7 show no significant difference in hematocrit and PLT volume fraction distribution.

## Discussion

**Figure 8:**
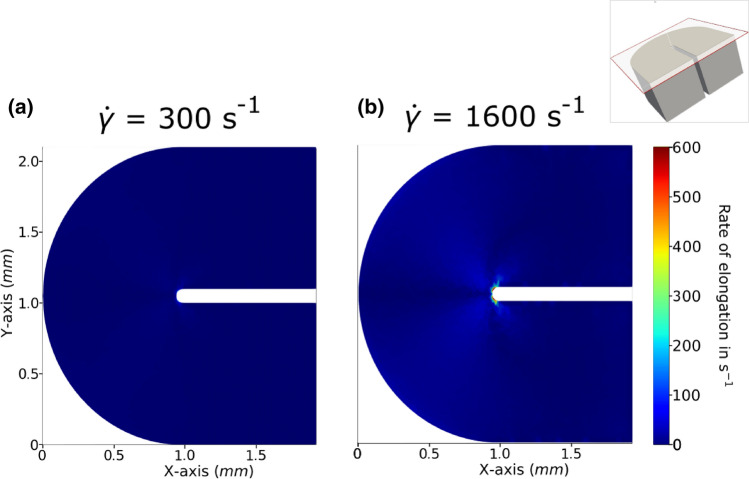
Elongational flow magnitude across 1 mm diameter U-channel in continuum simulation. 3D continuum simulation using the finite element method software FreeFEM (version 4.8, Sorbonne University, Paris, France) resembling a parallel plate flow chamber with Newtonian fluid and a dynamic viscosity $\upnu ~=~3.5$ Pas across a U-channel with 1 mm channel diameter and 100 ${\upmu }{\mathrm{m}}$ inner arc diameter. Boundary conditions are the same as in the cellular (HemoCell) simulations. The magnitude of the diagonal elements of the rate of strain tensor in flow dimensions produces the 2D elongation profile of the (a) 300 s^−1^ and (b) 1600 s^−1^ initial $\hbox {WSR}$ case. The rate of elongation reaches peak values of ${\dot{\varepsilon }}$ = 134 s^−1^ in the ${\dot{\gamma }}$ = 300 s^−1^ case and ${\dot{\varepsilon }}$ = 715 s^−1^ in the ${\dot{\gamma }}$ = 1600 s^−1^ case. To allow for better comparison to the results in Fig. 6, the same scale is used. The plane is situated 1 *μ*m below the top boundary of the geometry in z-direction, as depicted in the top right inset panel.

The effects of channel curvature on $\hbox {CFL}$ width and cross-sectional flow profile and cellular distributions are investigated, as well as their strongly interconnected relationships. Although, based on the large $180^\circ$ curvature of the channel, changes in overall cell distribution might be expected, no such significant deviations in $\hbox {CFL}$ width and $\hbox {PLT}$ concentration distributions are observed between the inner and outer arc of the curvature (see Figs. 3 and 7). In contrast to this, a strong compression of the shear rate profile towards the curve can be seen, resulting in a discrepancy in the $\hbox {WSR}$ ratio between the inner and outer wall of approximately 2:1 (see e.g. Fig. 4e). These observations are true for the comparatively small simulated channel. To strengthen transferability to the experimental results in a much larger channel, simplified Newtonian fluid continuum simulations are performed at dimensions of the $\hbox {MFD}$. The results (see Fig. 8) reveal qualitative similarity in the macroscopic continuum quantities to the cellular simulations (see Fig. 6).

Although cellular distributions in blood flow and width of the $\hbox {CFL}$ are known to be highly shear rate dependent,^1^ the observed strong shift in shear rate appears to be associated with changes only in the bulk flow, away from the walls. This might imply changes in the local $\hbox {PLT}$ margination process through the shift in local hematocrit gradients, however in the presented case, the $\hbox {PLTs}$ are already fully marginated by the time they enter the domain of observation in the simulations. The situation is assumed to be the same for the $\hbox {PLT}$ in the *in vivo* blood experiments, due to the inlet length of the blood perfusion tube and perfusion time of 4 minutes until the microscopic images are taken. As shown in Fig. 2, the results for the ${\dot{\gamma }}$ = 1600 s^−1^ flow experiment present a highly amplified PLT adhesion intensity at the collagen coated surface of the inner arc, with a 10% increase in thrombi coverage compared to the inlet region. This behaviour cannot be attributed to an increased availability of $\hbox {PLTs}$ in the observed region, considering the static size of the $\hbox {CFL}$ and the already fully accomplished margination. The shift in hematocrit concentration towards the inner wall of the curvature (see Fig. 7) cannot explain the increased adhesion either since it occurs in both high and low flow velocity cases and can be assumed to be situated at the center of the channel, due to having no significant influence on the $\hbox {CFL}$ width.

One could argue that due to a larger volumetric flow rate at the inner arc of the curve compared to the outer arc, simply more $\hbox {PLTs}$ pass by the observed surface region, therefore allowing for adhesion to occur more frequently. While a shift of the profile towards the curvature in plot B of Fig. 4 does show, approximately the same relative shift is visible in the low shear simulation (Fig. 5b). However, comparing the ${\dot{\gamma }}$ = 1600 s^−1^ and ${\dot{\gamma }}$ = 300 s^−1^ *in vivo* results (Fig. 2c), only the high shear experiment exhibits an increase in thrombi coverage at the inner wall of the curved section. Consequently the discrepancy cannot be explained solely by an increased volumetric flow in the region.

The multimeric plasma protein $\hbox {vWF}$ is a key mediator in the adhesion and aggregation of $\hbox {PLTs}$.^11^ Multiple domains of the protein enable binding to different molecules. Of special interest in our investigation are the A1 domain with binding sites for $\hbox {PLT receptor glycoprotein (GP) Ib}\upalpha$ of the GPIb-V-IX complex and A3 which allows for binding to collagen.^28^ Under physiological flow conditions, circulating plasma $\mathrm{vWF}$ is folded and unable to expose its A1 domain which allows interaction with the GPIb-IX complex. After vessel injury, $\mathrm{vWF}$ becomes adsorbed through its A3 domain, to subendothelial proteins, notably collagen, exposed to the flowing blood. Immobilized $\mathrm{vWF}$ experiencing shear flow (especially $> 1000$ s^−1^), unfolds and exposes the A1 domain, thereby supporting PLT attachment through GPIb-IX.^7,28^ As these necessary conditions are available throughout the microfluidic channel (in the ${\dot{\gamma }}$ = 1600 s^−1^ case) they do not explain the increased adhesion at the inner arc of the curve. Explosive PLT adhesion, as it occurs in acute arterial stenosis and resulting in thrombosis, is associated with pathological shear rates above 5000 s^−1^ and therefore known as shear-induced PLT adhesion. Here, free flowing $\mathrm{vWF}$ molecules uncoil while not yet bound to a substrate.^21^ This has also been reported to be caused by the same interaction at the surface of activated PLTs within a thrombus, highlighting a key role of the GPIb-IX/$\mathrm{vWF}$ bond formation in thrombus growth.^5^ In our simulated results peak $\hbox {WSRs}$ stay below 2700 s^−1^ and therefore such pathological shear rates do not occur. Shear gradients, as observed in Figs. 4 and 5e, are known to drive PLT adhesion.^15^ Sing and Alexander-Katz exposed that the underlying factor are elongational flows, which naturally occur in the presence of shear gradients. These flow fields play a key role in the adhesion mediation of vWF, by enabling vWF to unfold already in physiological shear flow conditions.^23^

**Figure 9:**
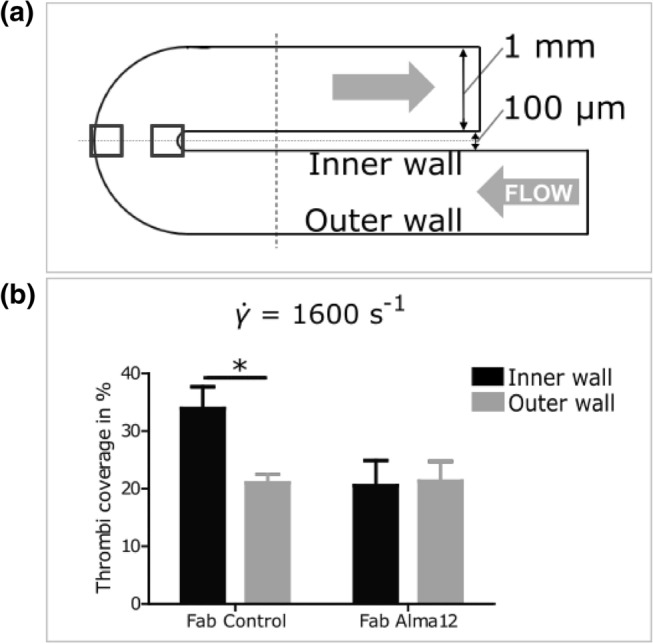
Impact of Fab ALMA12 preincubation on PLT aggregation to collagen in curved flow chamber. Hirudinated human whole blood is preincubated with fragment ALMA12 and subsequently perfused through channels of a MFD coated with a solution of type I fibrillar collagen (200 $\upmu$ g/mL). (a) Schematic and dimensions of the microfluidic “U-shaped” channel. The squares indicate the regions of interest observed by video-microscopy. (b) The bar graph represents the quantified surface coverage of platelet aggregates obtained after 4 minutes of perfusion at ${\dot{\gamma }}$ = 1600 s^−1^ with ALMA12 and control fragment. The bars indicate the mean ± $\hbox {SEM}$ thrombi coverage in the 2 highlighted regions of 5 separate experiments performed with different blood donors.

Based on the simulation strong elongational flows are observed at the inner arc of the channel’s curvature at a WSR of 1600 s^−1^ (see Fig. 6a), which correlates well with the increased thrombi coverage in the same region and in the same shear flow. Furthermore, $\mathrm{vWF}$ molecules that unfold in presence of the enabling flow conditions and do not immediately come in contact with a binding site are in a position to stay unfolded further into the flow, even when the rate of elongation falls below the initially enabling value.^25^ The increased error margin in thrombi coverage at the high shear case outlet section might hint at this effect, though a more detailed investigation is necessary. The elongation rate measured at the inner arc of larger than 370 s^−1^ is well within the critical rate ${\dot{\varepsilon }}_{c}$ (300–600 s^−1^) allowing for ${\mathrm{vWF}}$ molecules to uncoil in elongational flow.^23^ In accordance with our assumption, the low shear flow case exhibits a substantially lower rate of elongation at the inner arc peaking at 71 s^−1^, which is below ${\dot{\varepsilon }}_{c}$. This hypothesis will have to be confirmed in upcoming experiments in which the effect of pharmacological anti-PLT agents blocking the GPIb-IX complex, the A1 domain of $\mathrm{vWF}$ or other PLT receptors will be evaluated to proof dependence on the shear sensitive $\mathrm{vWF}$ molecule.^20^ Preliminary experiments with the Fab ALMA12 blocking agent seem to confirm this by cancelling out the difference in thrombi coverage between inner and outer wall at the channel curvature (see Fig. 9). Furthermore, while simplified 3D Newtonian fluid continuum simulations of the larger geometry already reveal qualitatively similar results (see Fig. 8), approximating the microfluidic chamber geometry by up-scaling the simulated domain in HemoCell will allow for a more quantitative transfer of the *in-silico* observations on the experimental results. Additionally, investigating the precise mechanical force which the shear flow applies on PLTs, vWF molecules and the wall could be suited better to compare between the collagen-bound and the plasma $\hbox {vWF}$ mediated adhesion.^8^

The current work presents the foundation for a deeper understanding of cellular flow behaviour in non-trivial curved geometries, as most blood vessels are, and their implications on $\hbox {PLT}$ adhesion.
